# Angiosarcoma of the Breast with Solitary Metastasis to the Ovary during Pregnancy: An Uncommon Pattern of Metastatic Disease

**DOI:** 10.1155/2013/209610

**Published:** 2013-12-07

**Authors:** Jayson Wang, Cyril Fisher, Khin Thway

**Affiliations:** ^1^Sarcoma Unit, Royal Marsden Hospital, London SW3 6JJ, UK; ^2^Department of Histopathology, Royal Marsden Hospital NHS Foundation Trust, 203 Fulham Road, London SW3 6JJ, UK

## Abstract

Primary *de novo* angiosarcoma of the breast is an uncommon, aggressive neoplasm. Here, we present a case of a young woman who initially developed primary angiosarcoma of the breast, and subsequently angiosarcoma of the ovary during pregnancy two years later. Only two confirmed primary angiosarcomas of the breast metastasizing specifically to the ovary have been described in the literature. However, all previous cases had ovarian metastases at presentation or shortly after initial diagnosis. This case is unusual as it occurred after a relatively long interval, and apparently developed during pregnancy. We discuss this rare phenomenon, as well as the possible factors contributing to the recurrence.

## 1. Introduction

Angiosarcomas are rare, usually aggressive soft tissue neoplasms, originating from endothelial cells. They are most frequently encountered in the skin (usually in sun-exposed sites or areas of vascular stasis), breast, and soft tissues. In the breast, angiosarcoma accounts for <0.1% of all malignancies, although is one of the commonest sarcomas at this site [1]. It often presents following radiotherapy for breast cancer, usually after an interval of several years [2]. Primary *de novo* angiosarcoma of the breast is rare [3] and usually seen in relatively young women in the child bearing age group [4]. Up to half of angiosarcomas are associated with metastatic disease, either at presentation or developing subsequently [5], and several case series have shown that these tumors most often metastasize to the liver, lung, or bones [6, 7]. Here, we present a case of a young woman who presented with primary angiosarcoma of the breast and who developed further angiosarcoma of the ovary during pregnancy two years later. This is an exceptionally rare phenomenon that we discuss in further detail.

## 2. Case Report

**Figure 1:**
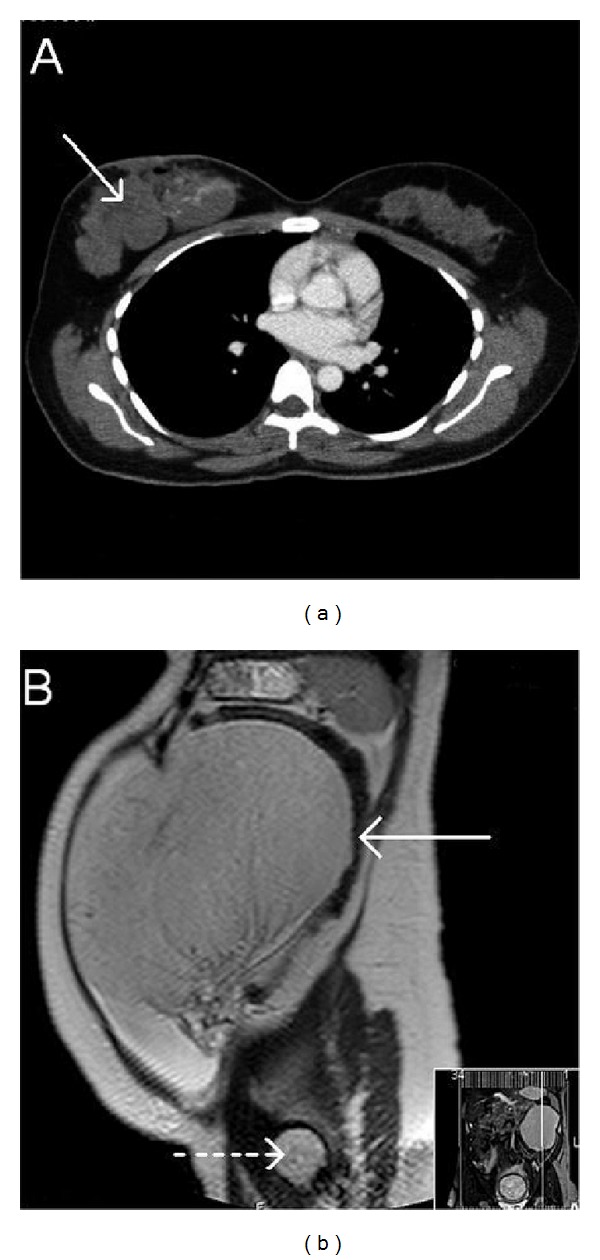
(a) Computed tomography (CT) scan show multifocal masses in the deep parenchyma of the right breast (arrowed); the largest measuring 4.6 cm in maximum diameter. (b) Magnetic resonance imaging (MRI) scan showed a large 19 × 17 × 8.3 cm smooth, circumscribed homogeneous solid mass in the left upper quadrant (arrowed), which was seen to displace the gravid uterine fundus to the left of the midline (dotted arrow), without mural invasion.

A 34-year-old female presented with an enlarging mass in the right breast. She had no relevant past medical history of note. She underwent a lumpectomy in her local hospital in a different country, and histology showed angiosarcoma, with positive margins. Computed tomography (CT) scan showed multifocal masses in the deep parenchyma of the right breast, the largest measuring 4.6 cm in maximum diameter (Figure 1(a)). The chest and abdomen showed no other disease. She was referred to our institution, where she proceeded to right completion mastectomy, with postoperative radiotherapy. She remained well for two years, at which time she was found to have an abdominal mass in the third trimester of pregnancy. Magnetic resonance imaging (MRI) scan showed a large 19 × 17 × 8.3 cm smooth, circumscribed solid mass in the left upper quadrant (Figure 1(b)). This lesion showed internal vascularity but was homogeneous in texture and was seen to displace the gravid uterine fundus to the left of the midline, without mural invasion, and there was no local peritoneal infiltration. No other abdominal disease foci were identified. Radiologically, the features were unusual for metastatic angiosarcoma and were more suggestive of a lymphoproliferative disorder. At 36 weeks' pregnancy the patient underwent Caesarean section, at which it was noted that the tumor seemed to be arising from the left ovary. Placental findings were normal. She subsequently underwent laparotomy and resection of the ovarian mass, which was thickly encapsulated with a thin reniform shape, possibly secondary to compression between the uterus and the undersurface of the left hemidiaphragm. The mass had a smooth and even surface, without any apparent tumor on the peritoneal surface. It was seen to replace the entire left ovary and was excised easily with the left fallopian tube. The uterus, right ovary, and all other intra-abdominal organs were normal, and no other tumor foci were identified surgically.

## 3. Materials and Methods

Immunohistochemical staining (streptavidin-biotin peroxidase complex method, with diaminobenzidine as the chromogen) was performed on formalin-fixed paraffin-embedded (FFPE) tumor tissue using a panel of commercial antibodies.

## 4. Results

### 4.1. Pathology

**Figure 2:**
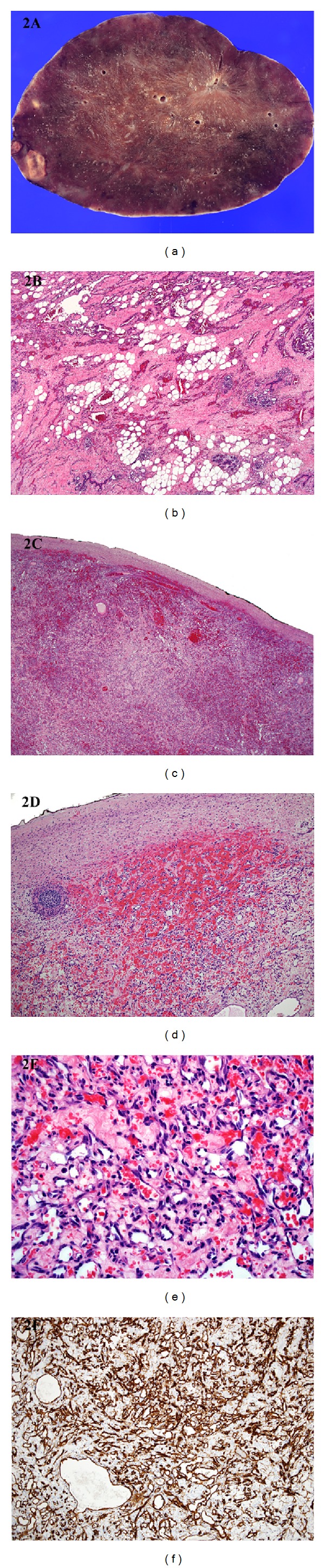
(a) Gross photograph of a transverse section of the large 14 × 10 × 6 cm ovarian mass. This lesion is seen to essentially replace the entire ovary and is composed of fleshy, deep red tissue. The ovarian surface is smooth and the capsule is grossly intact. There are areas of pallor and an area of necrosis at the left. (b) Histology from the previous mastectomy specimen shows extensive angiosarcoma within the deep breast parenchyma comprising irregularly anastomosing vascular channels lined by spindle cells with hyperchromatic nuclei. There is some preservation of breast lobular units (bottom right of field). (c) The subsequent oophorectomy specimen shows almost complete effacement of the ovarian parenchyma by similar angiosarcoma. Note the completely intact capsule and absence of tumor on the ovarian surface. (d) Only tiny amounts of peripheral ovarian parenchyma remain: a thin rim of ovarian tissue is seen peripherally and an intact follicle is seen on the left. The tumor is well differentiated and extensively vasoformative, with formation of blood lakes. (e) At higher power, the vascular channels are lined by mildly to moderately atypical spindle cells. (f) The cells are diffusely positive for CD34.

The mastectomy specimen comprised right breast and axilla weighing 550 g and measuring 15 × 14 × 5 cm, with an attached ellipse of nipple-bearing skin. Gross sectioning showed a poorly defined 6.5 × 4 × 7 cm hemorrhagic brown tumor lying 2 cm deep to the nipple and predominantly in the upper inner quadrant. The subsequent ovarian lesion consisted of a large, deep red 14 × 10 × 6 cm solid ovoid mass with smooth, intact capsule, and with no discernible surface tumor (Figure 2(a)). The 5 × 1 cm fallopian tube was attached at one side. Slicing revealed a homogeneous, medium firm, dark red/brown cut surface with focal areas of pallor up to 1 cm in diameter each, possibly representing necrosis.

Histologically, both the wide excision and mastectomy specimens showed extensive, invasive vasoformative tumor centred predominantly within breast tissue (Figure 2(b)), with focal extension into the deep dermis. Irregular anastomosing vascular channels were lined by spindle cells with minimally to mildly atypical hyperchromatic nuclei. There were occasional more solid cellular areas containing pleomorphic epithelioid cells. Blood lakes were prominent, and there was focal infarction and incipient necrosis. The mitotic index was up to 14/10 hpf. The surrounding breast parenchyma showed foci of fat necrosis with relative preservation of breast lobular units. The tumor showed diffuse and strong positivity for CD31, CD34, FLI1, and INI1, with smooth muscle actin (SMA) positive surrounding smooth muscle layers around most vessels. The tumor was negative for D2-40, HHV8, desmin, AE1/AE3, MNF116, and CAM5.2. The appearances were consistent with angiosarcoma, which was 1.3 mm from the posterior margin of the mastectomy specimen. Five reactive lymph nodes were present.

The subsequent oophorectomy specimen showed tiny amounts of peripheral ovarian parenchyma extensively replaced by similar neoplasm (Figures 2(c) and 2(d)), comprising mildly to moderately pleomorphic spindle cells forming anastomosing vascular channels with focal solid areas (Figure 2(e)). There was focal necrosis and a mitotic index of 3/10 hpf. The tumor focally infiltrated the parametrium, but the surrounding capsule was microscopically largely intact. The fallopian tube was uninvolved. The tumor was diffusely positive for CD31, CD34 (Figure 2(f)), and ERG, with SMA-positive smooth muscle seen focally around vessel walls, and negative for AE1/AE3, S100 protein, desmin, and HHV8. MIB1 labelled approximately 5% of tumor nuclei. The morphology was essentially identical to that seen in the previous breast excision specimens and was consistent with angiosarcoma. The patient made a good postoperative recovery and is being followed up, and remained well ten weeks after excision of the ovarian mass.

## 5. Discussion

After cutaneous neoplasms occurring in the head and neck region, the breast is the next most common site of origin of angiosarcomas. Of these, the majority arise secondary to adjuvant breast and chest wall radiotherapy, following wide local excision or mastectomy for breast carcinoma. It is thought that radiotherapy alone causes a sixfold increase in risk of angiosarcoma, with combined radiotherapy and chemotherapy increasing the risk to 100-fold (without increase in risk seen with chemotherapy alone) [2]. The interval between therapy and incidence of secondary angiosarcoma typically ranges from 1 to >20 years (mean 7 years) [8, 9]. Thus, radiotherapy-induced angiosarcoma usually affects older women, with a median age of 55–66 years. By comparison, primary angiosarcomas of the breast affect a younger cohort of women, with a median age of 35–42 [4] and tend to form palpable, frequently deep masses in contrast to the ill-defined or multifocal cutaneous patches or nodules in secondary cases. Furthermore, many reported primary angiosarcomas appeared to be well differentiated, with histological features sometimes mimicking benign lesions, with absence of atypia or mitoses, being shown to be malignant only after subsequent metastasis or death. However, both primary and secondary angiosarcomas have a high rate of recurrence and metastasis, with similar survival rates [6–9]. Grading angiosarcoma is not considered useful, as grade is not associated with prognosis [10].

Large series of angiosarcomas of the breast appear to show similar patterns of spread, regardless of whether tumor is a primary neoplasm or occurs secondary to radiotherapy [4, 6, 7]. The commonest sites of spread, apart from locoregional recurrences, are lung, bone, and liver. Most metastases arise from hematogenous dissemination, and nodal metastases are relatively uncommon. The occurrence of metastasis of angiosarcoma specifically or predominantly to the ovary is rare. While Chen et al. authored a case review claiming that metastasis to the ovary was a common site [11], no details of these cases were provided in the report. Many of the cases quoted in the literature are from the early 20th century, before immunohistochemical evidence was available, and many reports are not in the English literature. Of the verifiable reports of ovarian metastasis, all were found at postmortem in the context of widespread disseminated disease [12, 13]. To our knowledge, there have been only four confirmed previous cases of breast angiosarcoma metastasizing predominantly to the ovary [14–17]. All the breast tumors were primary, without history of radiotherapy, and occurred in women of childbearing age. Of these, one patient presented with synchronous bilateral ovarian tumors as well as splenic metastasis [15], while two others had had prior breast surgery and presented with a unilateral ovarian mass a few months later [14, 17]. In one of the cases, metastasis developed within an ovarian cyst. One last report showed breast angiosarcoma metastatic to the ovary and placenta during pregnancy, but the case details were not retrievable [16].

Angiosarcoma metastatic to the ovary is therefore exceptional, in contrast to other neoplasms that are well recognized to metastasize to the ovary, including adenocarcinoma from the gastrointestinal tract and lobular carcinoma of the breast. With regard to metastasis of sarcomas to the ovary, Young and Scully studied 21 cases [18], of which the commonest primary was from the uterus, particularly leiomyosarcoma, with the remainder from the gastrointestinal tract. In contrast to angiosarcoma of the breast, primary ovarian angiosarcoma is relatively rare, with the largest series describing seven cases [19]. Angiosarcomas of the ovary may be pure or admixed with (or can arise from) other neoplasms, commonly teratomas, as well as adenocarcinomas and fibromas [20–23]. Although there is a wide age range, similarly to primary breast angiosarcomas, most patients with ovarian angiosarcomas present at child bearing age (albeit the upper limit, with a median age of 42) [19, 24]. Many patients have had previous pregnancies, and one presented shortly after childbirth [24]. The prognosis is usually poor, with patients progressing to metastatic disease, including in the peritoneum, liver, lung, and bones.

Given the rarity of primary ovarian angiosarcoma and the chronology of the clinical findings in this current case, it seems less likely that this could represent an (initially occult) ovarian primary which first manifested clinically as a soft tissue metastasis to the breast. One reason this warrants consideration is because of the unusual surgical and gross histological findings of an ovarian mass with intact and smooth capsule, with tumor essentially contained within the ovarian capsule and stroma, without any evidence of surface tumor seeding, which might be expected in angiosarcoma metastatic to the ovary and in this case suggesting that the neoplasm grew within the organ. This tumor was also unilateral and ovarian metastases (at least for carcinomas) often occur bilaterally [25], although our review showed two previous cases of unilateral ovarian metastasis of angiosarcoma, described above. In our case, there had been no prior evidence of an ovarian mass, and this had not been detected at earlier fetal abnormality scanning, making the possibility of primary ovarian angiosarcoma with breast metastasis remote. Furthermore, we have not been able to identify from the literature any definite cases of ovarian angiosarcoma metastatic to the breast. It is not possible to exclude the remote possibility that the ovarian tumor represented a second primary angiosarcoma. As the ovarian hilum is richly vascular, seeding of the tumor can arise from this central location. Several conditions are known to predispose to angiosarcomas, such as Klippel-Trenaunay-Weber and Maffucci syndromes, which may lead to multifocal or multicentric disease [26, 27], but there was no evidence of these syndromes in our patient.

Regardless of the primary site in our case, an interesting observation is that both primary angiosarcomas of breast and ovary tend to occur in younger women and may be associated with prior or recent pregnancies, which has been commented on even in the early literature [28]. However, our case is only the second report of a metastasis related to pregnancy [16], and there has been only one further possible case of presumed “metastasis” of angiosarcoma developing in a pregnant woman; however, she presented with intracranial angiosarcoma, with no primary site found; thus a primary central nervous system angiosarcoma remained a possibility [29]. Although some studies have found estrogen and progestogen receptor expression in angiosarcomas [30], we, as well as others, have not found their expression in these neoplasms [31]. Regardless, hormonal or cytokine influences on the pathogenesis of these tumors should be considered, and further research into this area is warranted.

In summary, we present a case of a young woman who presented with primary breast angiosarcoma, followed by a unilateral ovarian angiosarcoma two years later during pregnancy. This highlights an exceptional pattern of metastatic disease, and raises the possibility that both primary and recurrent angiosarcomas may be, in some way, hormonally driven.
